# Haemophilia and Fragility Fractures: From Pathogenesis to Multidisciplinary Approach

**DOI:** 10.3390/ijms24119395

**Published:** 2023-05-28

**Authors:** Angelo Alito, Federica Bellone, Simona Portaro, Giulia Leonardi, Vittorio Cannavò, Francesca Coppini, Danilo Leonetti, Antonino Catalano, Giovanni Squadrito, Domenico Fenga

**Affiliations:** 1 Department of Biomedical, Dental Sciences and Morphological and Functional Images, University of Messina, 98100 Messina, Italy; alitoa@unime.it (A.A.); danilo.leonetti@unime.it (D.L.); 2 Department of Clinical and Experimental Medicine, University of Messina, Via Consolare Valeria, 1, 98100 Messina, Italy; vittorio.cannavo@hotmail.it (V.C.); catalano.antonino@unime.it (A.C.); giovanni.squadrito@unime.it (G.S.); 3 Department of Physical and Rehabilitation Medicine, University Hospital “G. Martino”, 98100 Messina, Italy; simonaportaro@hotmail.it (S.P.); giulia.leonardi@polime.it (G.L.); 4 Department of Orthopaedics and Traumatology, University Hospital A.O.U. “G. Martino”, 98100 Messina, Italy; francesca.coppini1@gmail.com (F.C.); dfenga@gmail.com (D.F.)

**Keywords:** fragility fractures, haemophilia, multidisciplinary approach, rehabilitation, secondary osteoporosis

## Abstract

Haemophilia A (HA) and haemophilia B (HB) are X-linked inherited bleeding disorders caused by the absence or deficiency of coagulation factors VIII (FVIII) and IX (FIX), respectively. Recent advances in the development of effective treatments for haemophilia have led to a significant increase in life expectancy. As a result, the incidence of some comorbidities, including fragility fractures, has increased in people with haemophilia (PWH). The aim of our research was to perform a review of the literature investigating the pathogenesis and multidisciplinary management of fractures in PWH. The PubMed, Scopus and Cochrane Library databases were searched to identify original research articles, meta-analyses, and scientific reviews on fragility fractures in PWH. The mechanism underlying bone loss in PWH is multifactorial and includes recurrent joint bleeding, reduced physical activity with consequent reduction in mechanical load, nutritional deficiencies (particularly vitamin D), and FVIII and FIX deficiency. Pharmacological treatment of fractures in PWH includes antiresorptive, anabolic and dual action drugs. When conservative management is not possible, surgery is the preferred option, particularly in severe arthropathy, and rehabilitation is a key component in restoring function and maintaining mobility. Appropriate multidisciplinary fracture management and an adapted and tailored rehabilitation pathway are essential to improve the quality of life of PWH and prevent long-term complications. Further clinical trials are needed to improve the management of fractures in PWH.

## 1. Introduction

Haemophilia A (HA) and haemophilia B (HB) are rare, inherited, recessive, X-linked bleeding disorders caused by the absence or deficiency of coagulation factors VIII (FVIII) and IX (FIX), respectively [1]. A third rare form is haemophilia C, which is associated with a deficiency of clotting factor XI [2].

**Figure 1:**
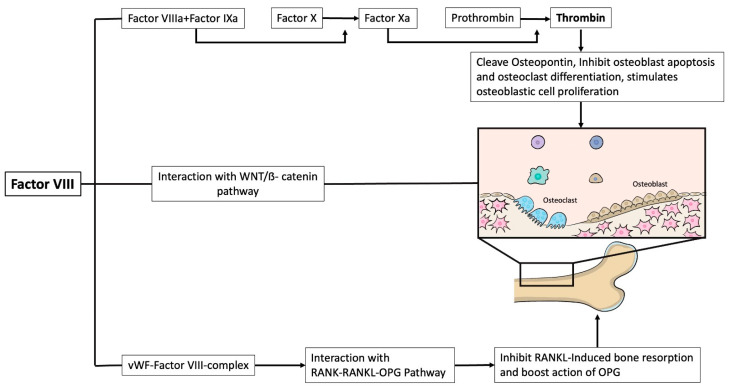
Influence of blood clotting factors on bone homeostasis.

HA is the most common form (85%) with a prevalence of about 1:5000 in male live births [3]. Conversely, HB occurs in approximately 15% of people with haemophilia (PWH), with an estimated prevalence at birth of 1:30,000 in males [3]. Advances in the development of effective and safe treatments over the last 50 years and their regular and widespread availability have led to a significant increase in the patients’ life expectancy [4]. The management of this new cohort of middle-aged and older patients is challenging due to the conflicting risks of haemophilia and age-related cardiovascular disease, malignancies, and comorbidities [5]. In addition, haemophilia-related comorbidities are more prevalent with increasing average age and life expectancy of patients [4]. Over the past 20 years, several studies have shown an increased risk of low bone mineral density (BMD) in PWH, even in children, despite similar levels of bone mineral content (BMC) and serum vitamin D compared to the general population [6]. From childhood, PWH rarely engage in weight-bearing activities due to the fear of bleeding and chronic pain caused by haemophilic arthropathy, resulting in failing to achieve adequate bone mass and affecting the relative risk of fracture [7]. The link between bone disease and haemophilia has been further investigated using FVIII knockout (KO) mice: despite the absence of haemophilic arthropathy, a reduced bone mass and strength due to an increased rate of bone resorption was observed [8]. In addition, FIX knockout mice showed a similar bone phenotype, suggesting a relevant role of thrombin signalling in bone remodelling [9]. The absence of FVIII or FIX and the failure to activate FX results in a deficient production of thrombin, the latter being able to cleave osteopontin, which is required for osteoclast anchoring to the mineralized matrix [10]. Furthermore, thrombin inhibits osteoblast apoptosis and osteoclast differentiation and stimulates osteoblastic cell proliferation, thereby improving bone formation and reducing bone resorption [11]. The role of FVIII is not limited to activation of the coagulation pathway; it also appears to be a biological regulator of bone metabolism [12]. FVIII binds to circulating von Willebrand factor (vWF) to prevent its rapid degradation [13]. Recent in vitro studies have shown that the FVIII–vWF complex plays an important role in bone health through its interaction with the receptor activator of NF-κB (RANK), its ligand (RANKL) and the osteoprotegerin (OPG) signalling pathway (RANK-RANKL-OPG). Specifically, this complex inhibits RANKL-induced bone resorption and enhances the action of OPG [14]. FVIII–vWF complex deficiency leads to increased osteoclastogenesis and bone loss, which in turn increases the risk of fracture [14]. The balance between bone formation and resorption is closely linked to the canonical Wnt/β-catenin signalling pathway, yet there are limited data on its involvement in PWH [15]. The influence of blood coagulation factors on bone homeostasis is shown schematically in Figure 1. In addition, sclerostin, which is a member of the Dickkopf (Dkk) protein family, acts as an antagonist of the Wnt/β-catenin pathway by binding to LDL Receptor-Related Protein 5 (LRP5) and LRP6 on the cell membrane of osteoblasts, thereby reducing osteoblast-mediated bone formation [15]. Low serum levels of sclerostin and Dkk-1 have been found in adult PWH [15]. There are conflicting data on the relationship between sclerostin levels and the severity of haemophilia [16]. In a recent study by Anagnostis et al., patients with severe haemophilia had lower sclerostin concentrations than those with mild or moderate disease [17]. This may be partly explained by the relatively older mean age of the sample, although in another study the enrolled PWH patients were younger (puberty), and sclerostin levels were positively associated with arthropathy scores [18].

Three distinct HA and HB phenotypes can be distinguished based on the reduction in serum FVIII or FIX levels: severe (<1 IU/dL), moderate (1–5 IU/dL) and mild (5–40 IU/dL) [19]. Nowadays, the replacement therapy has led to PWH having life expectancy like that of age-matched patients with comorbidities [20]. 

PWH, particularly those with the untreated severe forms, frequently exhibit recurrent bleeding in various organs [5] and, in particular, the musculoskeletal system [21]. 

Bleeding occurs in the major synovial joints, such as the ankles, knees and elbows, and its repetitive nature is widely recognized as key to the development of haemophilic arthropathy [22]. Several authors recognize inflammatory synovial changes as the main cause of bleeding because of the high vascularization and friability of the blood vessels, which reduces the ability to drain blood away from the joint [23,24]. This leads to hyperplasia of the synovial membrane with deposition of haemosiderin that perpetuates the cycle, resulting in the production of inflammatory mediators that cause cartilage and focal bone damage and joint remodelling [25,26]. In addition, several studies have shown that subclinical and untreated joint bleeding may be a factor in the worsening of arthropathy [27]. Repeated episodes of haemorrhage during growth lead to restriction of movement, limitation of weight-bearing exercise and fear of pain and re-bleeding, resulting in a reduction in achievable peak bone mass (PBM) [28]. As a result, reduced bone mineral density (BMD) and impaired bone microarchitecture lead to an increased risk of fragility fractures in PWH [29]. However, the pathogenesis of osteoporosis in PWH is not yet fully elucidated and includes thrombin deficiency [30], altered osteoblast and osteoclast activity [31] and reduced mobility or prolonged immobilization [12].

Treatment of osteoporosis in PWH includes calcium and vitamin D supplementation. Several options have been proposed: both antiresorptive and osteoanabolic drugs may be considered, such as bisphosphonates and denosumab, selective estrogen receptor modulators (SERMs) and teriparatide, although the use of these agents should be avoided in young PWH [32].

Overall, osteoporosis is recognized as a serious problem for PWH, since a four-fold increased fracture risk has been reported in comparison with the healthy population [33,34].

Advances in the management of PWH have led to significant improvements in clinical outcomes and quality of life, but these patients continue to suffer from bone fragility and high fracture-related mortality [35]. Because of the peculiar pathophysiology of bone fragility in PWH, which could be associated with both reduced bone formation and increased bone resorption, patients may benefit from both anabolic agents (teriparatide or neutralising antibodies against Dkk-1 or sclerostin such as romosozumab) [36,37] and antiresorptive agents, such as bisphosphonates or denosumab (human monoclonal antibody to RANKL) [38]. Curiously, serum sclerostin levels were significantly elevated in studies of children with severe HA [18]; therefore, romosozumab, a specific anti-sclerostin antibody that inhibits sclerostin-LRP5/6 interaction and that indirectly activates canonical Wnt signalling pathways and bone formation, may be effective in the pharmacological treatment of osteoporosis in PWH. However, further clinical trials are needed to evaluate the efficacy and safety in this specific cohort [39]. Recent studies have even shown a statistically significant difference in 25(OH)D_3_ and DEXA Z-scores between patients receiving twice-weekly FVIII prophylaxis (15 U/kg/dose) and those receiving on-demand therapy, suggesting a possible bone benefit of prophylaxis in PWH [40]. Therefore, by preventing bone and muscle damage and possibly reducing fracture rates, vitamin D supplementation may counteract the vicious circle that leads to reduced mobility in PWH [41]. With regard to the research studies investigating anti-osteoporotic agents in PWH, only one work is currently available on the efficacy of ibandronate, but data on fracture risk reduction are lacking. Administration of this amino-bisphosphonate for 12 months to 10 PWH resulted in an increase in lumbar spine BMD of 4.7%, but no significant change in hip or femoral neck BMD [42]. The limited data on the use of denosumab in PWH by Lin et al. documented an improved fracture healing in a man whose BMD increased significantly after 4 months of teriparatide followed by 1 year of denosumab treatment [12]. Therefore, regarding teriparatide, an anabolic agent, the effects on bone health of PWH are poorly understood to date [39]. 

Alongside causes closely related to clotting alterations, several mechanisms have been proposed to contribute to the development of fragility fractures in PWH, including nutritional deficiencies [43], impaired joint ROM [44], reduced muscle strength [42], balance deficits [45], HIV or HCV infection [31], and impaired biomechanics [46].

These risk factors predisposing to reduced bone mineral density are not exclusive to haemophilia but are a specific determinant of reduced bone mineralisation in many comorbidities associated with osteopenia/osteoporosis [47]. Therefore, appropriate multidisciplinary fracture management and an adapted and tailored rehabilitation pathway are essential to improve the quality of life of PWH and prevent long-term complications [48,49,50]. Rehabilitation is a key component in restoring function and maintaining mobility, while avoiding bleeding episodes or muscle trauma during rehabilitation exercises that delay patient recovery [51]. 

The aim of this narrative review is to summarize the pathogenesis of osteoporosis, analyse the causes and incidence of fragility fractures, and explore the need for a multidisciplinary approach in PWH.

## 2. Search Strategy

A comprehensive literature search was conducted to identify published studies on the management of fragility fractures in PWH. Three researchers independently conducted the review using the same keywords. Finally, papers were selected by consensus. The databases PubMed, Scopus and Cochrane Library were searched. The following string was used (haemophilia OR haemophilia) AND (fracture OR fragility fracture). Identified articles were screened using the following inclusion criteria: (i) study design: randomised controlled trials (RCTs), reviews, mini reviews, original articles, (ii) written in English, (iii) published in the last 20 years (2003–2023) in indexed journals, and (iv) dealing with fracture management in PWH. Exclusion criteria were drug use, animal studies, radiological studies, and other article types such as letters to the editor, case reports, editorials, and conference abstracts. Ethical approval was not required due to the study setting. Articles were screened first by title and abstract and then by full text analysis. The following data were collected: (1) study design; (2) patient characteristics; (3) fracture incidence; and (4) association between haemophilia and fractures.

**Figure 2:**
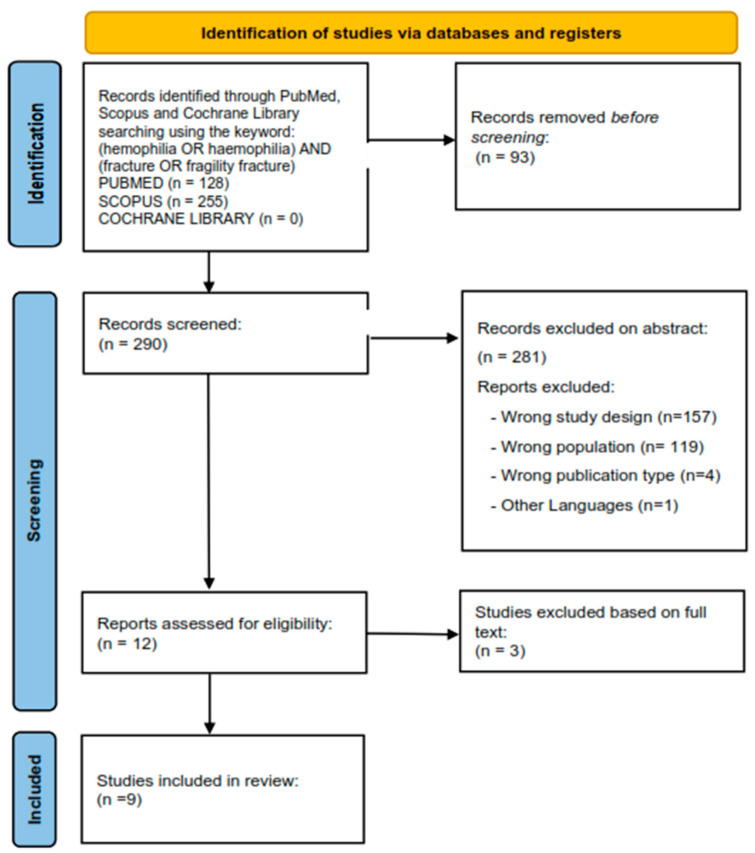
Preferred Reporting Items for Systematic Reviews and Meta-analyses flowchart of the paper selection process.

A flowchart of the process is shown in Figure 2. The initial search yielded 383 articles (PubMed, 128; Scopus, 255, Cochrane Library: 0). Duplicate articles were excluded. After evaluation of the inclusion and exclusion criteria, nine studies were included in this review.

## 3. Evidence of Fractures in Haemophilia

The authors analysed articles analysing the genesis and risk of fracture in PWH, considering age and gender. The articles included in this review are listed in Table 1. All of the analysed studies showed that PWH are associated with a reduction in BMD and an increased risk of fracture. No data about biochemical profile, gonadal status and calciotropic status at baseline were provided in the papers considered. Data about fracture incidence was only driven historically and not by the performance of imaging. In their retrospective analysis, Gay et al. analysed 382 male patients with HA and HB and compared them with a large cohort of the general healthy population matched for age and gender. They found that the risk of fracture increased with disease severity and age (with the risk doubling after the age of 31), with an increase of about 1.3% per year of age [52]. 

Anagnostis and colleagues highlighted in their review that bone loss in PWH begins in childhood, suggesting the need for a multidisciplinary approach to prevent further bone loss with regular assessment of fracture risk [28]. 

In their cohort study of 75 patients, Tuan et al. noted the late diagnosis of osteoporosis in PWH, more often reached at the time of the first fragility fracture. They also found that the incidence of osteoporotic fractures in haemophilia patients was significantly higher than in the general population. The authors also suggested a long follow-up period, given the chronic nature of the disease [32]. 

In their paper, Lee et al. studied 11 HA patients with intracapsular femoral fractures and showed that these fractures occurred almost 20 years earlier in PWH in comparison with healthy individuals. They also suggested a standard post-operative management (i.e., early mobilization and verticalization if possible) with the need for adequate haemostasis [53].

Pai et al. performed a 14-year cohort study showing a higher risk of fragility fracture with a no effect in all sites of fracture and repeated fractures [35]. 

Angelini and colleagues emphasized the importance of managing chronic pain and preventing falls and fractures in older PWH considering the typical comorbidities of ageing [54,55]. 

Finally, in 2015, a study by Caviglia et al. reported that osteoporotic fractures due to low-energy or overuse trauma in PWH are more common in younger people of the same age. In addition, with the advent of replacement therapy, there has been a reversal in the trend of lower and upper limb fractures, with a reduction in the average age [56].

**Table 1:** Data extracted from included studies.

Publication	Study Design	Patient Features	Fracture Incidence	Results	Correlations between Haemophilia and Fractures
Pai et al., 2022 [35]	Retrospective study (compared the incidence of all-site fractures, repeated fractures and osteoporotic fractures occurring in all PWH)	9152 (832 vs. 8320) Age: <20 years 4576 (50.00%) 20–39 years 3135 (34.25%) 40–64 years 1210 (13.22%) ≥65 years 231 (2.52%) Sex: NA	The incidence of fractures in PWH is inconclusive, and no research has yet explored repeated fractures among PWH.	Screening, prevention and treatment of osteoporosis and further osteoporotic fractures among PWH remain essential in order to improve quality of life and achieve healthy aging in this particular population.	PWH had a higher risk of osteoporotic fracture, but the haemophilia only had a neutral effect in all sites of fracture and repeated fractures.
Tuan et al., 2019 [32]	Nationwide population-based cohort study based on the data in the Taiwan National Health Insurance Research Database (PWH vs. control patients without haemophilia)	375 (75 vs. 300) Age: 35.7 (11.2–48.3) vs. 35.7(11.2–48.3) Sex: M:45 F: 30 vs. M:120 F:180	The cumulative incidence was significantly higher for PWH diagnosed more than 5 years	Strong association between haemophilia and the development of osteoporotic fractures after haemophilia diagnosis. The relative risk of osteoporotic fracture after haemophilia in PWH increased with age in those aged <65 years compared with non-PWH. Clinicians should pay particular attention to osteoporotic fractures after haemophilia in PWH as they age.	PWH have significantly lower bone mineral density. Lower bone mineral density may lead to fragile in-bone structure and even osteoporotic fractures.
Angelini et al., 2016 [54]	Review (focused on common complications affecting the older haemophilia population, including joint disease, cardiovascular disease, malignancy, renal insufficiency and liver disease)	Comparison of age distribution of PWH between 2011 and 2015 (Age <45 vs. Age 45–64 vs. Age >65) Sex: NA	Markedly increased risk of fracture in PWH, which corresponding with both disease severity and increasing age	The elderly PWH must cope with chronic joint arthropathy, which provokes falls and fractures, and complications related to HIV and HCV infections, which greatly impact the incidence of cancer and liver disease.	Aside from functional impairment, PWH are also at higher risk of fractures secondary to decreased bone mineral density.
Strauss et al., 2016 [57]	Retrospective study (PWH with surgical fracture fixation compared to a matched non-haemophilic control group)	46 fractures after low-energy trauma in 44 PWH vs. 46 non-haemophilic patients Age: 42.4 ± 20.5 years (range, 7–85 years) vs. 43.8 ± 20.7 years (range, 14–84 years) Sex: M: 38 F: 6 vs. M:23 F: 23	Higher incidence of fractures in PWH compared to a population without haemophilia	There was no significant difference regarding the duration of the preoperative hospital stay between PWH and controls.	End-stage haemophilic arthropathy, muscle atrophy and joint contracture on the one hand increase the risk of falling and on the other hand lead to osteoporosis, making the bone more susceptible to fracture after trivial trauma.
Anagnostis et al., 2015 [28]	Review (on the current understanding of the association between haemophilia A or B and low bone mass, as well as the optimal approach and management of bone disease in these patients)	Age: NA Sex: NA	Data for increased fracture risk in PWH are currently not robust because of the rarity of the disease and the patients’ relatively young age; it can be speculated that it should be higher than in the general population	Regular exercise, prophylactic factor replacement therapy for severe haemophilia, fall prevention strategies and optimising calcium and vitamin D intake are recommended.	Except for low bone mineral density, PWH seem to be at increased fracture risk. Individualized multidisciplinary approach and careful assessment and management of risk factors associated with increased fracture risk are recommended.
Angelini et al., 2015 [55]	Review (focus on common complications affecting the older PWH, including cardiovascular disease, malignancy, liver disease, renal insufficiency, and joint disease)	Age: mean age 54.5 years (range 31 to 72) vs. mean age 46.7 years (range 23 to 76) Sex: M: 11 F: 18	Higher incidence of fractures in PWH	Elderly PWHs should be treated similarly to their peers without haemophilia, with the addition of factor replacement therapy as appropriate. Primary prevention of risk factors should be emphasized, and close coordination between specialties is essential	In the older PWH, chronic joint arthropathy provokes falls and thus an increased fracture risk
Caviglia et al., 2015 [56]	Retrospective study (on 28 years’ experience treating PWH who suffered fractures and evaluating the impact of access to treatment)	151 fractures in 141 PWH (25 vs. 35 vs. 33 vs. 31 vs. 27) Age: NA Sex: NA	Higher incidence of LL fractures in the first period analysed (1986–1990); over time, the ratio LL/UL changed as UL fractures became more frequent. This change is due access to treatment and specifically to the prophylaxis.	Fractures in PWH became more common in the UL than in the LL, lowering the age at which they occur and being less frequent. The advent of new and accessible treatments decreased the development of orthopedic complications and favours improvement in the quality of life of PWH.	Daily activities for PWH are reduced due to the impact of multiple bleeding episodes in the musculoskeletal system, leading to arthropathy and contractures. In addition, muscle wasting, osteoporosis, joint stiffness and malalignment can increase the risk of fractures.
Gay et al., 2015 [52]	Retrospective study (on increased fracture rates in PWH: a 10-year single institution retrospective analysis)	382 patients (316 with haemophilia A vs. 66 with haemophilia B) Age: 20 (±18) vs. 26 (±21) Sex: NA	PWH had an increased fracture rate compared with the control population. PWH with mild to moderate haemophilia had a significantly reduced risk of fracture compared with those with severe disease.	Increased fracture risk with increasing severity of haemophilia, coupled with the evidence for bleeding-independent mechanisms of decreased skeletal health, suggest factor replacement may directly impact bone health and fracture risk in PWH.	PWH have a higher risk of reduced bone mineral density than the general population. It is currently unclear how this predilection for reduced bone mineral density translates into fracture rates.
Lee et al., 2007 [53]	Case report (comprehensive report on the management of a cohort of patients with fracture of neck of femur in haemophilia)	11 Age: mean age was 30 years (range: 16–55) Sex: NA	In PWH, most of the femoral neck fractures (9 out of 11) are seen almost two decades earlier than in general population (where they occur in patients over 50 years of age)	In PWH, femoral neck fractures can be treated as in the general population, with modest dose of factor replacement. Postoperatively, prolonged use of plaster immobilization should be avoided and early mobilization of the ipsilateral knee joint should be initiated.	Although physical activity is often reduced in PWH, poor musculature, osteoporosis and haemophilic bone changes may predispose to an increased risk of fractures.

NA= Not Available.

## 4. Discussion

Modern replacement therapies have increased the average life expectancy of PWH, as well as the incidence of chronic diseases (e.g., diabetes, stroke, cardiovascular disease, renal failure, osteoarthritis) and the risk of falls and fractures [35,54]. Osteoporosis in PWH is also highly prevalent and multifactorial, but it is not always recognised as secondary osteoporosis [46]. Data in the literature agree that factor replacement therapy in PWH may directly impact bone health and fracture risk, supporting the already known role of FVIII and FIX in bone metabolism [52,58].

Several studies have shown that physical activity limitation during growth to prevent trauma, nutritional deficiencies and recurrent bleedings can lead to a reduction in BMD [12,58,59]. Most of the existing evidence has shown an association between low BMD and fractures only in children with haemophilia [60], not in adults [28].

Anagnostis et al. in 2012 assessed BMD using dual-energy X-ray absorptiometry (DXA) at the lumbar spine (LS), femoral neck (FN), total hip (TH) and greater trochanter (GT) in a cohort of adult PWH. They found low BMD in 26.9% of patients and 20% of controls (*p* = 0.0001). In detail, no differences were found in the mean values of BMD at the LS (1.047 ± 0.169 g/cm^2^ in PWH vs. 1.065 ± 0.156 g/cm^2^ in controls; *p* = 0.794), whereas a significantly lower BMD was found in PWH than in controls at the TH (0. 929 ± 0.161 g/cm^2^ and 1.018 ± 0.189 g/cm^2^, respectively; *p* = 0.007), FN (0.938 ± 0.163 g/cm^2^ and 1.015 ± 0.176 g/cm^2^, respectively; *p* = 0.029) and GT (0.757 ± 0.152 g/cm^2^ and 0.848 ± 0.167 g/cm^2^, respectively; *p* = 0.008) [61].

As described by Anagnostis et al., PWH show low BMD from an early age and appear to be at increased risk of minor trauma fractures and falls [28]. Reduced trabecular bone mineralization from early childhood, even in the absence of cortical damage, contributes to increased fragility [28]. To support the data on low BMD in PWH, Lee and colleagues performed a high-resolution peripheral quantitative computed tomography (HR-pQCT) analysis of 18 adult patients and found microarchitectural changes in cortical and trabecular bone compared with the general population [62]. Interestingly, Zang and colleagues reported a case of intra-articular femoral microfracture in which they observed increased osteoclast activity with reduced ability to repair damage due to the inflammatory environment [26]. These findings support a multifactorial genesis of bone fragility [5]. 

There is a direct correlation between the severity of haemophilia and changes in bone turnover homeostasis [59], with patients with severe haemophilia having a greater risk of fractures [52]. In addition, the development of target joints (at least three haemorrhages in 6 months) and the concomitant presence of muscle haemorrhages lead to reduced mobility and an increased risk of falls [58,63]. 

Furthermore, this arthropathy is not just about one joint—it has a widespread effect on bone metabolism and has a relationship with BMD [64]. So, it is often not just a question of avoiding high-risk activities, but also conditions such as fatigue, muscle weakness and contractures that restrict movement that contribute to the early development of osteoporosis [53]. 

Many authors have focused on the need for early assessment of BMD to allow primary prevention, as osteoporosis is often diagnosed after fracture occurrence [32,35,65]. The literature suggests that PWH have a higher risk of fracture than their peers, particularly those under 65 years or age, and that this may be explained by a combination of factors such as reduced previous physical activity, comorbidities, early bone loss and nutritional deficiencies [32,52,58]. 

Despite the interest in fragility fractures in PWH, there is no clear evidence of an increased fracture risk [58], with a surprising lack of increase in refracture risk likely explained by a reduction in physical activity [35].

Therefore, PWH should be monitored throughout the life course, as the incidence of fragility fractures in PWH appears to be higher 5 years after diagnosis, with a higher annual increase today [32]. It is interesting to note that the improvement in prophylactic treatment has changed the attitude of both patients and carers, allowing PWH to participate in outdoor activities and contact sports at a young age, with physical and psychological benefits [56]. This trend reversal led to an increase in the number of fractures in young PWH, particularly in the upper limbs [56]. These findings provide further evidence of the impact that replacement therapy has had over the last 50 years and the change in the quality of life of PWH [66]. 

Fracture management in PWH is the same as in the general population, with the aim of healing and restoring function, with surgery being the preferred option if conservative management is not possible [52,53,67,68]. Today, PWH with severe arthropathy or fractures requiring surgery (e.g., hip replacement) can be rehabilitated with less anxiety and improved outcomes, even with pre-operative physiotherapy programs [69]. The concept of rehabilitation for PWH has evolved over the last 30 years from a role in the bleeding phase only, to one of limiting disability, to one of prevention [70]. Management of bone disease in PWH should begin in childhood to achieve the best possible PBM, encouraging low-impact resistance training throughout life, regular assessment of BMD and fracture risk, and avoidance of smoking, alcohol consumption and obesity [28]. It is, therefore, important to remember that PWH are at increased risk of falling due to the combined effects of arthropathy, reduced muscle strength and balance problems [33], so, an exercise program focusing on strength, balance and motor coordination should be encouraged at all ages [71]. 

There are several limitations to this review, such as the unspecified fracture site and its effect on quality of life, few studies specifying the type of rehabilitation patients underwent, and the lack of randomised clinical trials. Assessment of fractures not by imaging techniques but by extrapolating them from medical record data and the absence of baseline evaluation of gonadal and calciotropic status further weaken this work. However, our research originally provides a deep explanation of mechanisms underlying fracture risk in PWH and gives an extensive view of the multidisciplinary fracture management in this setting of patients, leading to an improvement in quality of life and prevention of long-term complications.

## 5. Conclusions

In summary, this paper confirms the significant association between fracture risk and PWH, although it is difficult to correlate this with age. The results of this study suggest an association between age at fracture and haemophilia, and that haemophilia tends to occur at a younger age, with an average age of 25–30 years. In addition, the increased fracture risk in PWH appears to be due to a combination of factors that contribute to an increase in falls, such as comorbidities, balance, and motor impairments. On the other hand, younger PWH seem to be more prone to fractures because of the greater promotion of normal lifestyles that encourage exercise and physical activity. However, a fundamental role is certainly played by the ability of the bone to resist despite the weakening of its trabecular component and its poor acquisition of BMD. 

## 6. Future Directions

Young PWH have an increased susceptibility to fracture, probably due to a combination of factors such as repeated joint bleeding, reduced physical activity, and FVIII and FIX deficiency. However, the available literature does not give us confidence that these are the only reasons contributing to the pathogenesis of osteoporosis in PWH. Future studies could focus more on the quality of life and disability associated with different types of fractures.

## Data Availability

All data analysed in this study are included in this published article.
